# Relationship between appendicular muscular mass index and physical function in older people

**DOI:** 10.3934/publichealth.2024006

**Published:** 2024-01-11

**Authors:** Miguel Alarcón-Rivera, Carolina Cornejo-Mella, Camila Cáceres-Aravena, Yeny Concha-Cisternas, Paz Fernández-Valero, Eduardo Guzmán-Muñoz

**Affiliations:** 1 School of Sports Sciences and Physical Activity, Faculty of Health, Universidad Santo Tomás, Talca, Chile; 2 Faculty of Medicine, Universidad Católica del Maule, Talca, Chile; 3 School of Kinesiology, Faculty of Health, Universidad Santo Tomás, Talca, Chile; 4 School of Pedagogy in Physical Education, Faculty of Education, Universidad Autónoma de Chile, Talca, Chile; 5 Faculty of Human Sciences, School of Physical Education, Sports and Recreation, Universidad Bernardo O'Higgins, Santiago, Chile

**Keywords:** physical fitness, aged, postural balance, walking speed

## Abstract

This study aimed to establish the relationship between the appendicular muscle mass index (AMMI), assessed from anthropometric variables, and the physical function of older people. Seventy-six older people participated in this study (72.03 ± 7.03 years). The participants underwent evaluations to determine their AMMI using anthropometry (weight, calf circumference, hip circumference, and knee height) and manual grip strength. Additionally, their physical function was evaluated using the 5-chair stand test, the 3-meter walk test, and the timed up and go test (TUG) to determine the strength of the lower limbs, the gait speed, and the dynamic balance, respectively. The results show that the AMMI did not present a significant relationship with the 5-chair stand test in both women (*r* = -0.135; *p* = 0.204) and men (*r* = -0.067; *p* = 0.349). The AMMI was moderately correlated with the gait speed in both women (*r* = 0.542; *p* < 0.001) and men (*r* = 0.556; *p* < 0.001). Finally, a statistical significance was observed in the relationship between the AMMI and the TUG test in women (*r* = -0.273; *p* = 0.047) and older men evaluated in this study (*r* = -0.284; *p* = 0.042). In conclusion, there is a relationship between the AMMI and both the dynamic balance and the gait speed. Therefore, the AMMI emerges as a potential public health assessment by enabling the clinical quantification of muscle mass and an estimation of physical function in the elderly population.

## 1. Introduction

The increase in life expectancy worldwide has led to a steady increase in the number of people over 60 years of age [1]. It is projected that the percentage of the world's population over the age of 60 will nearly double from 12% to 22% between 2015 and 2050 [1]; in Chile, the older adult population will increase from 19.9% in 2017 to 21.6% in 2050 [2].

Aging is a biological, universal, individual, asynchronous, and natural process that causes morphophysiological changes in bodily systems [3]. At the muscular level, the loss of muscle mass and strength have been considered the main factors responsible for the deterioration of functionality in older people [4]. In fact, at present, the loss of muscle mass and strength are the main diagnostic criteria for the geriatric syndrome known as sarcopenia [5].

The appendicular muscle mass index (AMMI) is a measure of the muscle mass in the upper and lower limbs relative to height and can be calculated with indirect prediction models such as dual-energy X-ray absorptiometry (DXA), magnetic resonance imaging (MRI), computed tomography (CT) and bioimpedanciometry, as well as with double indirect models such as anthropometry [6]–[10].

Research has shown that AMMI measured by methods such as DXA, CT or bioimpedanciometry correlates with strength and function in the elderly [11],[12]. Tsukasaki et al. [12] compared the DXA and CT methods in the estimation of physical function variables such as grip strength, knee extension strength, leg extension power, and gait speed. These authors concluded that the correlation coefficient between physical function and the cross-sectional area (CSA) measured by CT was greater than the relationship found with the skeletal muscle mass derived by DXA for gait speed in men (*p* = 0.002) and knee extension strength in women (*p* = 0.03) [12]. Concerning bioimpedanciometry, a relationship has been reported between muscle mass and the knee extension strength (*p* < 0.01), though no relationship was found with the walking speed [11]. However, few studies have addressed this relationship with anthropometric methods. Understanding that evaluations by methods such as DXA, MRI, CT, and bioimpedanciometry in clinical practice are practically inaccessible and of a high economic cost [10], the assessment of the AMMI through anthropometric methods is of great interest.

Physical function is a critical part of the assessment of older people, both in clinical and research settings. To evaluate the physical function, traditional instruments measure the individual's ability to perform specific functional tasks such as walking or a chair stand [13]. Some of the most used tests to evaluate the physical function of older adults are the 5-chair stand test, the 3-meter walk test, and the timed up and go test (TUG), which measures the strength of the lower limb, the gait speed, and the dynamic balance, respectively [13],[14]. These assessments are clinically significant because of their ability to predict adverse health outcomes such as hospitalization, falls, institutionalization, disability, and mortality [13].

Given the relevance of physical function to the health of older people and the feasibility of evaluating muscle mass through anthropometric variables, this study aims to investigate the correlation between the AMMI and physical function in the elderly.

## 2. Materials and methods

### 2.1. Participants

This was a cross-sectional study. The sample participants were selected under accidental non-probabilistic sampling and consisted of a total of 76 older people (40 women and 36 men) from the Maule region of Chile. The inclusion criteria were as follows: a) individuals of both genders, aged between 65 and 80 years; b) those with a functional status indicating either self-employed or a self-employment risk as assessed by the Elderly Functional Assessment Measure (EFAM-Chile); and c) individuals scoring ≥14 points on the mini-mental test, signifying an optimal cognitive level. The exclusion criteria were as follows: a) being either institutionalized or hospitalized; b) using technical aids such as orthotics or prostheses for movement; c) participants diagnosed with vestibular disorders by a medical professional; d) those who had undergone surgical procedures within the six months preceding the study; and e) presence of pain, edema and/or inflammation at the time of evaluation.

#### 2.1.1. Ethics approval of research

All participants read and voluntarily signed an informed consent form based on the criteria and activities to which they were subjected. The research was approved by the ethics committee of the University of Santo Tomás, Chile (Number 18.20).

### 2.2. Protocols

#### 2.2.1. Appendicular muscle mass index

The AMMI of the Chilean older people was estimated using the equation proposed by Lera et al. (2014) [10]. The equation was used to estimate the appendicular muscle mass (AMM), which was later normalized by the height of the participants. The anthropometric methods proposed in the equation were weight, height, hip circumference, calf circumference, and knee height, which are detailed as follows:

AMM (kg) = 0.107 (weight in kg) + 0.251 (knee height in cm) + 0.197 (calf circumference in cm) + 0.047 (manual grip strength in kg) - 0.034 (hip circumference in cm) + 3.417 (male sex) - 0.020 (age in years) - 7.646.

Body weight was measured with a digital scale (Seca® Hamburg, Germany; precision of 1 kg). Height was assessed using a stadiometer (Seca® Hamburg, Germany; precision 0.1 cm) with the participant remaining in a bipedal position. To determine the hip and calf circumferences, an inextensible tape measure (Sanny®, Brazil; precision 0.1 mm) was used and the measurements were performed according to the protocol of the International Society for the Advancement of Kineanthropometry (ISAK) [15]. For the hip circumference, the participant had to be standing and the tape measure was placed parallel to the floor, at the level of the maximum circumference without compressing the soft tissues (buttocks). For the calf perimeter, the measurement was performed with the feet apart while distributing the weight on both feet, in which the maximum perimeter of the dominant leg was sought [15]. Knee height was measured using a wide-bladed knee caliper with the subject being seated; measurements were made on the left leg, placing the knee and leg at a 90-degree angle. The fixed part of the caliper was placed under the heel and the movable part was placed parallel to the fibula over the malleolus and immediately behind the fibula, thereby pressing the two blades together to compress the soft tissues [15].

Manual grip strength was assessed and included in the equation to determine the AMM. This test was evaluated using a dynamometer (Jamar® PC 5030 J1, Sammons Preston Rolyan, USA) on the dominant hand. The measurement was made with the participant seated, shoulders in abduction, elbow flexed at 90 degrees, with the forearm and wrist in a neutral position [16]. Then, they were asked to perform a maximal force with their dominant hand for 3 seconds, with a 1-minute rest between each repetition. Three measurements were taken, and the highest mark was recorded. Lera et al. (2014) [10] included the manual grip strength over other measures of physical function in their equation, since they considered it a strong predictor of mortality, disability, and institutionalization.

The result of the equation described above was normalized by dividing the AMM by the squared bipedal height, which corresponds to the AMMI; this final value was used in the analysis [10].

#### 2.2.2. Physical function

To assess the physical function of the elderly, we used the 5-chair stand-up and sit-down, 3-meter walk, and timed up and go (TUG) tests to determine the strength of the lower limb, the gait speed, and the dynamic balance, respectively [17]. The 5-chair stand-up and sit-down test (squats) consists of the person standing up and sitting down from a chair without armrests five times as fast as possible. The time (in seconds) it takes to perform the test is recorded [18]. The TUG test consists of measuring the time it takes the individual to stand up from a chair, walk three meters, turn around walk back to the chair, and then sit down again [19]. Finally, the 3-meter walk test measures the time (in seconds) it takes the participant to walk three meters in a straight line at a normal pace. The test is assessed by measuring the speed in meters/second. All tests were performed three times, and the shortest time achieved in each test was considered.

### 2.3. Statistical analysis

The statistical package for the social sciences (SPSS) statistical software, V25.0, analyzed data at an a priori significance level of <0.05. The results were described using measures of central tendency, such as mean and standard deviation. The Kolmogorov-Smirnov normality test was performed to determine the distribution of the data. A student's t-test for independent samples was performed to compare the characteristics of the participants age, weight, height, and body mass index (BMI). The Spearman's parametric test was used to determine the association between the AMMI and physical function. Based on the correlation coefficient (*r*-value), the relationships were interpreted as weak (0 to 0.39), moderate (0.4 to 0.69), and strong (0.7 to 1).

## 3. Results

Table 1 shows the mean and standard deviation of age, weight, height, and BMI of the participants in this study. No significant differences were observed in the characteristics of the participants (*p* > 0.05).

**Table 1.** Characteristics of the sample.

Project	Women		Men	
	*Mean*	*SD*	*Mean*	*SD*
Age (years)	72.03	7.03	72.62	7.75
Weight (kg)	70.10	13.10	76.15	11.14
Height (m)	1.54	0.04	1.66	0.06
BMI (kg/m^2^)	29.44	5.73	27.57	3.56

Note: BMI: body mass index; *SD*: Standard deviation.

Table 2 shows the results of the AMMI and the functional tests performed on both men and women evaluated in this study. The comparison of the AMMI (*p* < 0.001), 5-chair stand test (*p* < 0.001), gait speed (*p* < 0.001), and TUG (*p* < 0.001) revealed significant differences between men and women.

**Table 2.** Results of AMMI and functionality based on sex.

Project	Women		Men	
	*Mean*	*SD*	*Mean*	*SD*
AMMI (kg/m^2^)	6.53	1.15	7.76	0.84
5-chair test (s)	11.54	2.79	10.25	2.89
Gait speed (m/s)	0.79	0.26	0.90	0.17
TUG (s)	9.14	2.14	8.50	2.04

Note: AMMI: Appendicular muscle mass index; TUG: Time up and go test; *SD*: Standard deviation.

**Figure 1.**
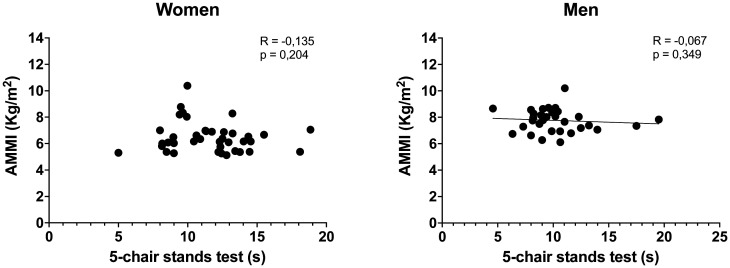
Correlation between AMMI and the number of repetitions in 5-chair stands test in women and men. In both sexes, the correlations were not significant based on sex (*p* > 0.05).

**Figure 2.**
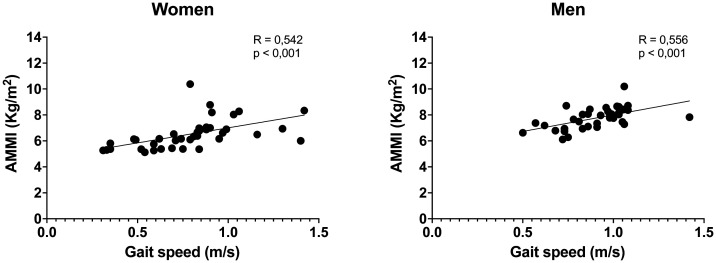
Correlation between AMMI and gait speed (m/s) in women and men. In both, the correlations were significant (*p* < 0.05), where it was observed that the higher the AMMI the higher the gait speed achieved by the elderly.

**Figure 3.**
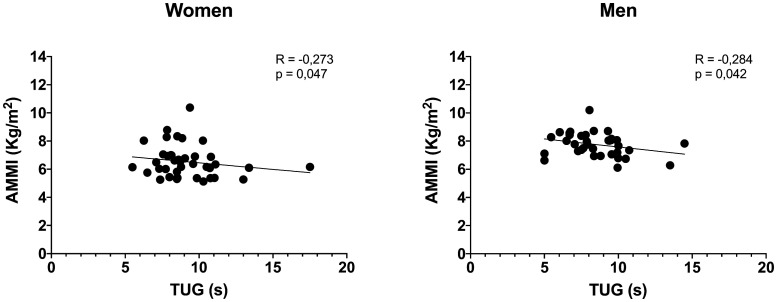
Correlation between AMMI and TUG test in women and men. In both, the correlations were significant (*p* < 0.05), in which it was observed that the higher the AMMI, the lower (faster) the TUG time.

The results of the correlations indicate that the AMMI did not present a significant relationship with the 5-chair stand test in both the women (*r* = -0.135; *p* = 0.204) and men (*r* = -0.067; *p* = 0.349) evaluated (Figure 1). For the gait speed, it could be observed that there was a moderate correlation with the AMMI in women (*r* = 0.542; *p* < 0.001) and men (*r* = 0.556; *p* < 0.001) (Figure 2). Likewise, a statistical significance could be observed in the relationship between the AMMI with the TUG test in the women (*r* = -0.273; *p* = 0.047) and older men evaluated in this study (*r* = -0.284; *p* = 0.042) (Figure 3).

## 4. Discussion

The objective of this study was to establish the relationship between the AMMI, assessed from anthropometric variables, and the physical function of older people. The results of our investigation indicate that there is a correlation between the AMMI and the physical function tests of gait speed and TUG in the older people evaluated. Similar to our findings, a positive correlation between muscle mass and gait speed has been previously observed [11],[20],[21]. Moreover, other studies has previously reported the relationship between muscle mass and the TUG test [20]–[22]. In addition, our research also revealed that there is no relationship between the AMMI and lower limb muscle strength assessed by the 5-chair stand test, which is a finding that contrasts with those found in previous studies [20]–[22]. Despite this, a trend is observed in our analysis, thereby suggesting that the higher the AMMI, the greater the muscle strength of the people evaluated in the squat test.

Some studies have shown that being able to get up from a chair is essential for independent living and that the performance of this action is strongly influenced by the ability of the lower limbs to exert a force by the muscle mass of the lower body [23]. Similarly, a study in an older Asian population found that in both men and women, knee extension strength showed significant positive correlations with the AMMI [11]. The discrepancy in the results obtained from other studies in the international literature could be attributed to the different evaluation methods used to obtain the AMMI (such as DXA, TC, and bioimpedanciometry), or to the different lower limb muscle strength assessment tests (such as 5-chair stand, knee extension, and manual grip) [11],[12]. While our research applied clinical tests to determine physical function, and a validated equation in the Chilean elderly population to obtain the AMM and the AMMI, other studies used objective but more expensive methods, such as DXA, MRI, or bioimpedanciometry [11],[24].

On the other hand, our research found a direct relationship between the AMMI and the gait speed test, in which people with greater muscle masses performed the test faster. Our findings are similar to those previously reported by Diaz-Villegas et al. [25] and Menant et al. [26], which found that muscle mass was a significant predictor of gait speed, independent of age and BMI [25],[26]. One possible explanation for these results is that a greater AMMI is associated with a greater functional independence and a better ability to perform daily life activities, such as walking and bending. In contrast, muscle weakness and atrophy of muscle fibers resulting from sarcopenia are associated with impaired physical function and higher rates of disability in older people [27]–[30].

In previous studies, the relationship found between muscle mass and physical condition were consistent in both sexes. For example, in both men and women, a significant relationship and similar correlation coefficients have been reported in tests such as lower extremity strength and hand grip strength [11],[12]. However, another study showed a relationship between muscle mass and gait speed only in men [12]. This differs from our findings, where we observed a moderate correlation in both sexes. The disparity in results might stem from our utilization of anthropometric estimates for assessing muscle mass, whereas other studies employed CT and DXA for measurements [12]. It has been postulated that gait speed could be considered the sixth vital sign to be evaluated in the elderly, since it correlates with parameters such as functional capacity, confidence, and balance, thereby allowing it to predict the future health status of individuals [31]. In this context, maintaining an adequate gait speed and an adequate muscle mass in the lower limbs is important to promote the functionality and autonomy of the elderly population [31].

Regarding the relationship between the AMMI and the TUG test, our results suggest that older people with a lower muscle strength in the lower extremities take longer to execute the TUG test. This finding is corroborated by previous studies, which have postulated that lower limb muscle mass, specifically weakness, is significantly related to the balance test performance [32].

Overall, our collective results are indicative of an age-associated deterioration in musculoskeletal function, as well as a substantial reduction in coordination and postural balance [3],[33]. These changes, along with alterations in sensory receptors, proprioception, visual, and vestibular function, impact postural control, the ability to maintain static and dynamic balance, and impair the ability to produce force, thereby increasing the risk of falling [3],[33].

As a secondary result, our research found significant differences in the AMMI between both sexes, in which men obtained a higher AMMI than women. Our finding coincides with what was previously reported by Bai et al. [34]. Regarding the functional tests, our study revealed that men presented better performances in the 5-chair stand, gait speed, and TUG tests, which is consistent with what was described by Okabe et al. [35]. This difference in physical function could be due to the higher AMMI reported in men and to the fact that the baseline characteristics of the participants (i.e., age, weight, height, and BMI) are homogeneous. Therefore, it is apparent that the main influencing factor on physical test performance was AMMI.

The strengths of our study are the measurement of physical function variables by means of nationally and internationally validated clinical tests, and the use of a valid, reliable, easy-to-obtain, and low-cost equation to predict the AMMI of older Chilean people. This undoubtedly positions it as a reproducible tool in clinical practice for identifying and screening people with low muscle mass and sarcopenia. In contrast, our research also has limitations, such as the small sample size and the convenience selection of participants, which restricts the external validity of the results and does not allow them to be generalized to the elderly Chilean population. Consequently, all conclusions from the results should be taken with caution.

## 5. Conclusions

The results of our research indicate that participants between 65 and 80 years of age with a higher AMMI present better functionality. Specifically, we observed a correlation between the AMMI and the gait speed and TUG in both women and men. Therefore, the AMMI emerges as a potential public health assessment by enabling the clinical quantification of muscle mass and an estimation of physical function in the elderly population. Additionally, we suggest their use in public health as a previously validated and reliable equation to predict the AMMI in older people. Likewise, it is easy assessment and low-cost clinical tool.

## Use of AI tools declaration

The authors declare they have not used Artificial Intelligence (AI) tools in the creation of this article.
